# Mitochondrial Defunctionalization Supresses Tim-3-Galectin-9 Secretory Pathway in Human Colorectal Cancer Cells and Thus Can Possibly Affect Tumor Immune Escape

**DOI:** 10.3389/fphar.2019.00342

**Published:** 2019-04-05

**Authors:** Svetlana S. Sakhnevych, Inna M. Yasinska, Elizaveta Fasler-Kan, Vadim V. Sumbayev

**Affiliations:** ^1^Medway School of Pharmacy, Universities of Kent and Greenwich, Chatham, United Kingdom; ^2^Department of Pediatric Surgery and Department of Biomedical Research, Children's Hospital, Inselspital, University of Bern, Bern, Switzerland; ^3^Department of Biomedicine, University Hospital Basel and University of Basel, Basel, Switzerland

**Keywords:** galectin-9, Tim-3, immune surveillance, mitochondria, colorectal cancer

## Abstract

The Tim-3-galectin-9 secretory pathway is known to protect various types of cancer cells against host immune surveillance. We found that pharmacologically induced mitochondrial dysfunction leads to a reduced galectin-9 expression/exocytosis in human colorectal cancer cells and re-distribution of this protein (the effect described for various cellular proteins) into mitochondria.

## Results

**Figure 1:**
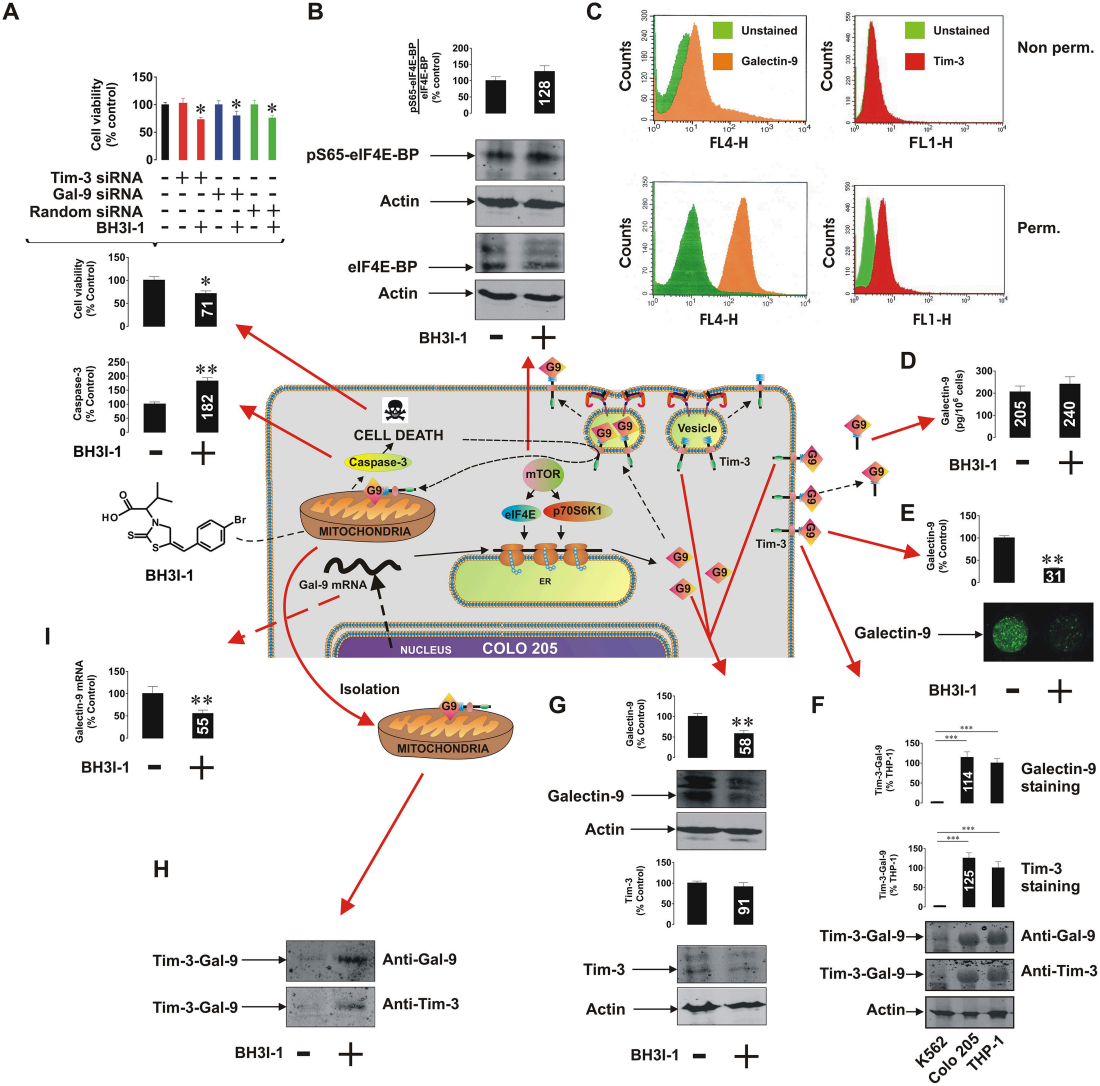
Pro-apoptotic defunctionalization of mitochondria reduces galectin-9 expression and leads to its redistribution in human Colo 205 colorectal adenocarcinoma cells. Colo 205 cells were exposed to 100 μM BH3I-1 for 24 h followed by **(A)** detection of cell viability using an MTS test and colorimetric assay of caspase-3 activity. Cell viability was also tested for normal and Tim-3 or galectin-9 knockdown Colo 205 cells. **(B)** Following 24 h of exposure to BH3I-1 S65-phosphorylation levels of eIF4E-BP were analyzed by Western blot. **(C)** Surface presence and total cellular levels of Tim-3 and galectin-9 were analyzed in Colo 205 cells using FACS. **(D)** Secreted levels of galectin-9 were analyzed in Colo 205 cells following 24 h of exposure to BH3I-1 by ELISA. **(E)** Surface levels of galectin-9 in non-treated and BH3I-1-stimulated Colo 205 cells were compared using an on-cell assay. **(F)** The presence of Tim-3-galectin-9 complex in Colo 205 cells was confirmed using Western blot analysis (bands were appearing at around 70 KDa, better detectable when temperature denaturation is not applied). THP-1 cells were used as a positive and K562 as a negative control. **(G)** Levels of Tim-3 and galectin-9 were analyzed in Colo 205 lysates following 24 h of exposure to BH3I-1 by Western blot. **(H)** Mitochondrial extracts were obtained from non-treated and BH3I-1-stimulated Colo 205 cells and subjected to Western blot analysis to detect Tim-3 and galectin-9. Total protein levels were measured using a Bradford assay and equal protein amounts were loaded onto the gels. **(I)** Galectin-9 mRNA levels were analyzed in non-treated Colo 205 cells and those exposed to BH3I-1 using qRT-PCR. In the scheme galectin-9 is abbreviated as G9. Quantitative results are shown as mean values (crucial mean values are written inside respective bars) ± SEM of 3–6 independent experiments. *p < 0.05; **p < 0.01; ***p < 0.001 vs. control. The scheme in the centre of the figure is based on our work on Tim-3-galectin-9 secretory pathway (Gonçalves Silva et al., 2017).

It has recently been discovered that the immune receptor Tim-3 (T cell immunoglobulin and mucin domain-containing protein 3) and it's ligand galectin-9 determines the capability of various types of malignant cells [e.g., acute myeloid leukemia (AML), colorectal cancer] to escape host immune surveillance (Kang et al., 2015; Gonçalves Silva et al., 2017; Sakhnevych et al., 2018; Yasinska et al., 2018b). Also, some of the galectin family members (for example galectin-3) were found to be able to protect AML and colorectal cancer cells against apoptosis through mitochondrial stabilization in a B cell lymphoma protein (Bcl) 2-dependent manner (Lee et al., 2013; Ruvolo, 2016). We asked whether galectin-9 has the same intracellular anti-apoptotic activity in addition to its extracellular immunosuppressive role. We used a pharmacological inhibitor 5-[(4-bromophenyl)methylene]-a-(1-methylethyl)-4-oxo-2-thioxo-3-thiazolidineacetic acid (BH3I-1, Figure 1A), a synthetic cell permeable Bcl-X_L_ antagonist, which induces apoptosis via inhibition of interactions between the BH3 domain and Bcl-X_L_ thus defunctionalyzing mitochondria. We found that BH3I-1 was capable of inducing apoptosis in Colo 205 colorectal adenocarcinoma cells of epithelial origin (based on increased caspase-3 activity and decreased viability of the cells, Figure 1A). Silencing either galectin-9 or its receptor and possible trafficker Tim-3 did not affect the pro-apoptotic activity of BH3I-1 suggesting that galectin-9 is unlikely to display anti-apoptotic activity in this case. Interestingly, the action of BH3I-1 did not affect the activity of mammalian target of rapamycin (mTOR) translational pathway as seen from its capability to phosphorylate eukaryotic initiation factor-4E-binding protein (eIF4E-BP, Figure 1B). Obviously, one could suggest that Colo 205 cells accumulate galectin-9 on their surface and inside the cells based on FACS analysis (Figure 1C). Reduced levels of surface-based Tim-3 might indicate its masking by galectin-9 (Yasinska et al., 2018a). BH3I-1 does not affect the ability of Colo 205 cells to secrete galectin-9 (Figure 1D) but significantly reduces its surface presence (Figure 1E) as measured by on-cell assay. Colo 205 cells accumulate the Tim-3-galectin-9 complex (Figure 1F) at a level comparable to THP-1 AML cells (K562 chronic ML cells expressing traces of galectin-9 were used as a negative control). Both proteins are also clearly detectable in Colo 205 cells by Western blot (Figure 1G) and treatment with BH3I-1 reduces intracellular levels of galectin-9. Importantly, Western blot analysis of Colo 205 mitochondrial extracts showed that the Tim-3-galectin-9 complex is accumulated in mitochondria upon stimulation with BH3I-1 (Figure 1H). The intracellular levels of galectin-9 mRNA were significantly reduced upon stimulation with BH3I-1, as detected by quantitative real-time PCR (qRT-PCR, Figure 1I).

Interestingly, the ability of Colo 205 cells to secrete galectin-9 is lower compared to THP-1 AML cells and the levels of secretion in both cell types are proportional to cellular Tim-3 levels (Supplementary Figure 1). This further supports conclusion regarding the involvement of Tim-3 in galectin-9 secretion (Gonçalves Silva et al., 2017).

We have also investigated two other types of epithelial cells—non-malignant human kidney RC-124 and malignant human HepG2 hepatoma cells. Both cell types have abundant mitochondria, however they are often [especially non-malignant, like RC-124—confirmed by a direct chemical measurement as described of the drug-associated bromine (Sollo et al., 1971) uptake in these cells, data not shown] less permeable for inhibitors of this type compared to colorectal cancer and AML cells. Therefore, 6 h of exposure to 1 mM H_2_O_2_ was used in order to defunctionalize mitochondria (Nicholas et al., 2011). We found that galectin-9 levels were significantly reduced in both cell types but the Tim-3-galectin-9 complex was only accumulated in the mitochondria of HepG2 and not RC-124 cells (Supplementary Figure 2).

## Materials and Methods

Commercially available Colo 205, RC-124, HepG2, THP-1, and K562, accompanied by authentication certificates, were used in this study. Mitochondria isolation, Western blot, on-cell assays, qRT-PCR, ELISA, and FACS analysis were performed as described before (Nicholas et al., 2011; Gonçalves Silva et al., 2016, 2017; Yasinska et al., 2018a). Detailed description of materials and methods used is provided in Supplementary Information.

## Discussion

Our results indicate that colorectal cancer cells operate the Tim-3-galectin-9 secretory pathway, where Tim-3 acts as a galectin-9 binding partner and possible trafficker. Pro-apoptotic mitochondrial dysfunction leads to a decreased transcription of galectin-9 mRNA leading to its reduced translation. However, exocytosis of galectin-9 is affected by mitochondrial defunctionalization leading to a re-distribution of the Tim-3-galectin-9 complex into mitochondria where galectin-9 could possibly interact with mitochondrial glycoproteins. The physiological relevance of this process is unclear but may well be a part of the regulated cell suicide programme which might involve transfer of galectin-9 into mitochondria so that it can't be involved in protection of a dying cell thus allowing its smooth elimination. Our further studies indicate that this phenomenon might be applicable mainly to malignant epithelial cells (Figure 1, Supplementary Figure 2). Importantly, targeted defunctionalization of mitochondria in malignant cells may be a novel strategy for anti-cancer immunotherapy since it reduces cell surface presence of galectin-9 capable of suppressing anti-cancer activity of cytotoxic lymphoid cells.

## Author Contributions

SS performed majority of the experiments reported in the Figure 1 and significant number of experiments reported in Supplementary Figures 1, 2, analyzed the data and contributed to manuscript writing. IY performed analysis of Tim-3-galectin-9 interactions and significant amount of experiments reported in Supplementary Figure 2, contributed to data analysis and manuscript writing. EF-K contributed to study design, performed FACS analysis, contributed to data analysis, and manuscript writing. VS designed the study, supervised the whole project, put the data together, wrote the manuscript.

### Conflict of Interest Statement

The authors declare that the research was conducted in the absence of any commercial or financial relationships that could be construed as a potential conflict of interest.
