# Supplementary material for: Mitochondrial Defunctionalization Supresses Tim-3-Galectin-9 Secretory Pathway in Human Colorectal Cancer Cells and Thus Can Possibly Affect Tumor Immune Escape
Source: Front Pharmacol. 2019 Apr 5;10:342. doi: 10.3389/fphar.2019.00342 (PMC6461007; doi:10.3389/fphar.2019.00342)
Supplement: Supplementary file 1 [file Table_1.docx]

**Mitochondrial Defunctionalisation Supresses Tim-3-Galectin-9 Secretory Pathway in Human Colorectal Cancer Cells and Thus Can Possibly Affect Tumour Immune Escape**

Svetlana S. Sakhnevych^1^, Inna M. Yasinska^1^, Elizaveta Fasler-Kan^2,3^* & Vadim V. Sumbayev^1^*

1 – Medway School of Pharmacy, Universities of Kent and Greenwich, Chatham Maritime, United Kingdom

2 - Department of Pediatric Surgery and Department of Biomedical Research (DBMR), Children’s Hospital, Inselspital, University of Bern, Bern, Switzerland

3 – Department of Biomedicine (DBM), University Hospital Basel and University of Basel, Basel, Switzerland


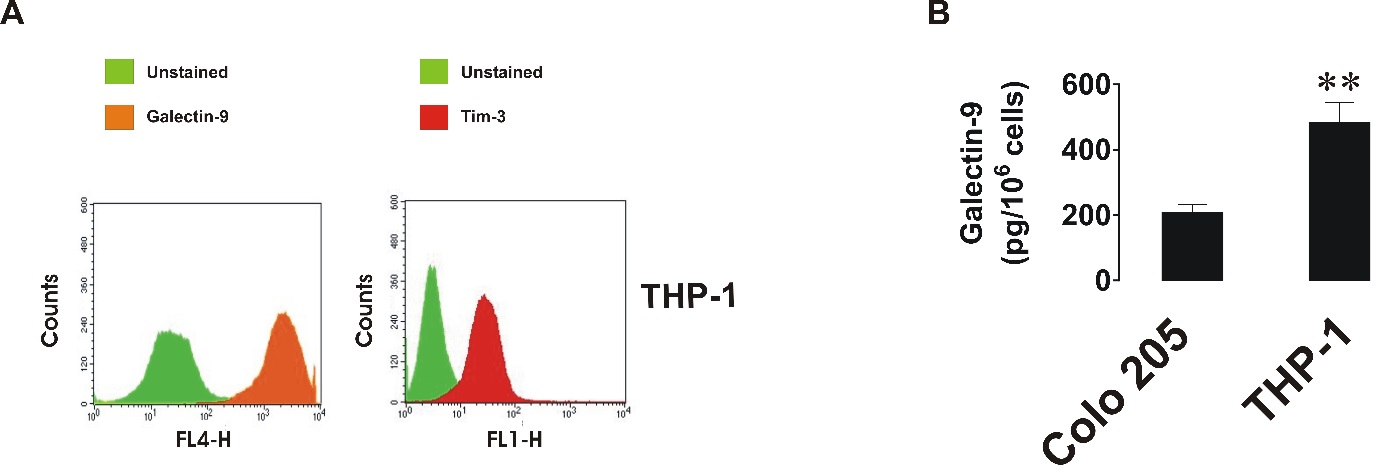


**Supplementary figure 1. Total cellular levels of Tim-3 and galectin-9 and levels of secreted galectin-9 in THP-1 human AML cells**. **(A)** Total Tim-3 and galectin-9 levels were measured by FACS in permeabilised THP-1 cells. **(B)** The secreted levels of galectin-9 were also measured in culture medium, in which THP-1 cells were kept for 16 h and compared with those of Colo 205 cells cultured under the same conditions. Images are from one experiment representative of four which gave similar results. Other results are shown as mean values ± SEM of five independent experiments. ** p<0.01 vs control.


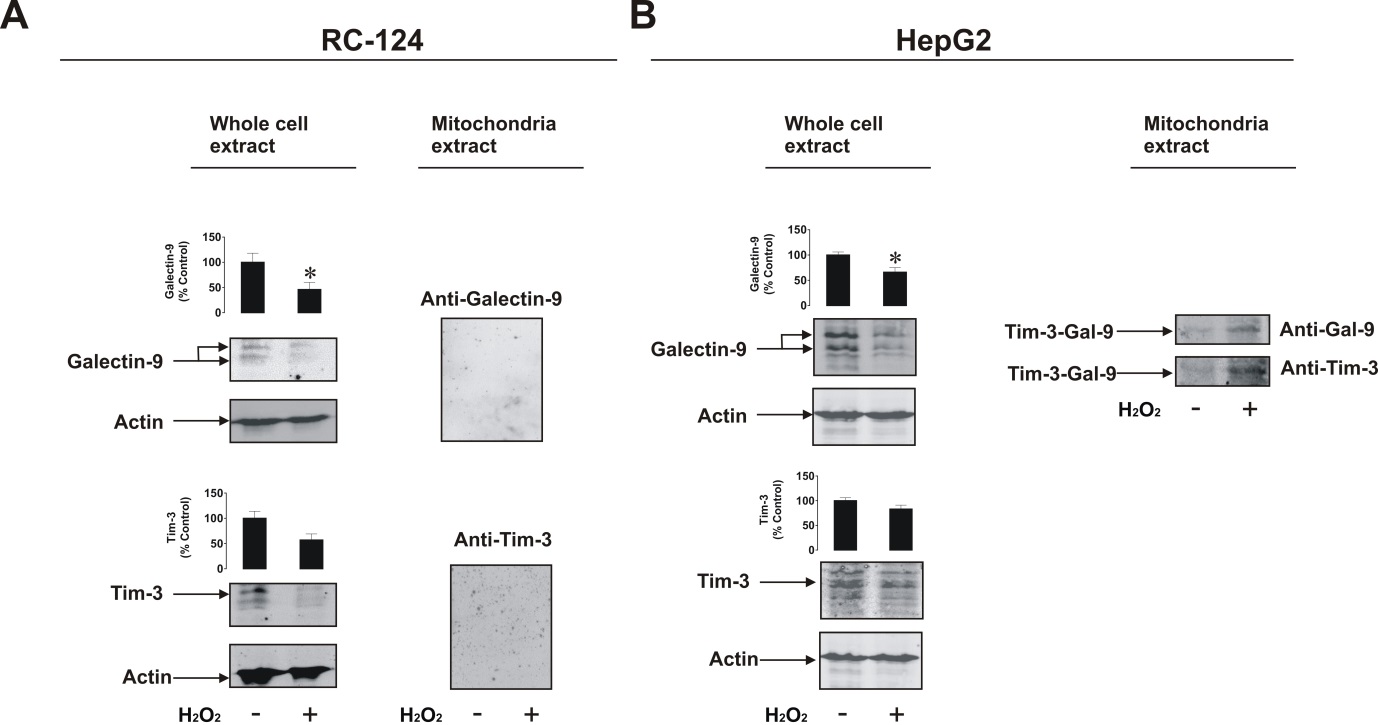


**Supplementary figure 2. Mitochondrial defunctionalisation reduces intracellular galectin-9 levels in both healthy (RC-124) and malignant (HepG2) epithelial cells but induces galectin-9 translocation into mitochondria only in malignant (HepG2) cells**. Cells were exposed to 1 mM H_2_O_2_ for 6 h followed by Western blot analysis of cellular and mitochondrial levels of Tim-3 and galectin-9 in both RC-124 **(A)** and HepG2 **(B)** cells. In mitochondria of RC-124 cells Tim-3, galectin-9 and the complex of both proteins (MW ~ 70 KDa) was not detectable, while in mitochondria isolated from HepG2 cells a complex was clearly detectable in H_2_O_2_-stimulated cells. Importantly, galectin-9 levels are higher in HepG2 cells (malignant) compared to RC-124 (non-malignant). This difference in fact may determine the effects observed. Images are from one experiment representative of at least four which gave similar results. Quantitative results are shown as mean values ± SEM of four independent experiments. ** p<0.01 vs control.

**Materials and Methods**

**Materials**

Tissue culture medium, foetal bovine serum and supplements as well as basic laboratory chemicals were purchased from Sigma (Suffolk, UK). Maxisorp™ microtitre plates were provided either by Nunc (Roskilde, Denmark) and Oxley Hughes Ltd (London, UK). Antibodies against phospho-S65 and total eIF4E-BP were purchased from Cell Signaling Technology (Danvers, MA USA). Goat anti-mouse/anti-rabbit fluorescence dye-labelled antibodies were obtained from Li-Cor (Lincoln, Nebraska USA). Anti-Tim-3 mouse monoclonal antibody and its single chain variant were produced as previously described (Gonçalves Silva et al., 2017). Rabbit anti-galectin-9 antibody was obtained from Abcam (Cambridge, UK). All other chemicals purchased were of the highest grade of purity and available commercially from Fisher Scientific (Loughborough, UK) and Sigma (Suffolk, UK).

**Cells and mitochondria isolation**

Commercially available cell lines obtained from European Collection of Authenticated Cell Cultures (ECACC, Salisbury, UK - Colo 205, HepG2, THP-1 and K562) or CLS Cell Lines Service GmbH (Eppelheim, Germany – RC-124) accompanied by authentication certificates, were used in this study. Cellular mitochondria were isolated using differential centrifugation (Nicholas et al., 2011). Cells were harvested followed by homogenisation in an isolation buffer containing 0.32 M sucrose, 1 mM EDTA (K+ salt), and 10 mM Tris–HCl (pH 7.4). Homogenates were subjected to centrifugation at 1330 × g for 3 min (procedure may need to be repeated twice depending on the cell type) and supernatants were centrifuged again at 21,200 × g for 10 min. Collected mitochondria can be further purified in a Percoll gradient. Protein content was analysed using the Bradford assay.

**Transfer of Galectin-9/Tim-3 siRNA into target cells and qRT-PCR**

We used galectin-9-specific siRNA target sequence (uga ggu gga agg cga ugu ggu ucc c) (Gonçalves Silva et al., 2016). For Tim-3 knockdown, we applied commercially available siRNA obtained from Santa Cruz Biotechnology, CA, USA (Gonçalves Silva et al., 2016). As a negative control, we used corresponding random siRNA (uac acc guu agc aga cac c dtdt) (Gonçalves Silva et al., 2016). Transfection of siRNAs into the target cells was performed using DOTAP reagent according to the manufacturer's instructions.

To monitor mRNA levels we used qRT-PCR (Gonçalves Silva et al., 2016). Total RNA was isolated by GenElute™ mammalian total RNA preparation kit. This was followed by reverse transcriptase–polymerase chain reaction (RT-PCR) of a target protein mRNA performed in accordance with the manufacturer's protocol (Sigma). Then quantitative real-time PCR was performed. Primer selection was the following: Galectin-9, 5′-CTTTCATCACCACCATTCTG-3′, 5′-ATGTGGAACCTCTGAGCACTG-3′ Tim-3, 5′-CATGTTTTCACATCTTCCC-3′, 5′-CTATGGCATTGCAAACGCACA-3′, actin, 5′-TGACGGGGTCACCCACACTGTGCCCATCTA-3′, 5′-CTAGAAGCATTTGCGGTCG-ACGATGGAGGG-3′. Reactions were performed using a LightCycler® 480 real-time PCR system and respective SYBR Green I Master kit (obtained from Roche, Burgess Hill, UK). Analysis was performed according to the manufacturer's protocol. Values representing galectin-9 and Tim-3 mRNA levels were normalised against β-actin.

**Western blot analysis**

Western blot analysis was performed as described earlier (Gonçalves Silva, et al., 2016 Gonçalves Silva, et al., 2017). β-actin staining was used to determine equal protein loading. Li-Cor goat secondary antibodies, conjugated with fluorescent dyes, were employed as recommended in manufacturer's protocol to visualise target proteins (Li-Cor Odyssey imaging system was used). Western blot data were quantitatively analysed using Odyssey software and values were subsequently normalised against those of β-actin.

In order to quantitatively analyse mTOR-dependent phosphorylation of eIF4E-BP (Yasinska et al., 2018a), we detected levels of phospho-S65-eIF4E-BP and total eIF4E-BP on different membranes to avoid the influence of possible incomplete membrane stripping. This was followed by quantitative analysis. Values were normalised against those of β-actin for respective membranes. The ratio between normalised phospho-S65-eIF4E-BP and total eIF4E-BP was calculated in order to characterise eIF4E-BP phosphorylation levels implementing the following equation.

$$\mathbf{pS65-eIF4E-BP level=}\frac{\left[ \mathbf{pS65-eIF4E-BP} \right]}{\left[ \mathbf{Actin} \right]}\boldsymbol{\div}\frac{\left[ \mathbf{eIF4E-BP total} \right]}{\left[ \mathbf{Actin} \right]}$$

This ratio in control samples was considered as 100%.

**On-cell assay, ELISA and FACS analysis**

We employed a standard Li-Cor on-cell assay to analyse surface presence of galectin-9 in target cells (Gonçalves Silva, et al., 2016). Levels of galectin-9 secreted into the medium were analysed by ELISA (R&D Systems assay kits) according to the manufacturer's protocol. For FACS analysis cells were fixed with freshly prepared 2% parafolmaldehyde, washed 3 times with phosphate buffered saline (PBS), permeabilised with 0.1% TX-100. Cells were incubated with an appropriate antibody overnight at 4°C or left unstained as a negative control ant then analysed using FACS Calibur cytometer with CellQuestPro software (Becton Dickinson, USA) as described (Yasinska et al., 2018b).

**Cell viability, caspase 3 activity and BH3I-1 uptake assays**

Cell viability was analysed using MTS assay kit (Promega) according to the manufacturer’s protocol. Caspase 3 activity assay was performed using colorimetric assay kit (R&D Systems). The method assumes use of specific substrate DEVD conjugated to p-nitroaniline. This allows detection of ability of caspase-3 to convert cleave the substrate in real time. The assay was performed in accordance with manufacturer’s instructions. Uptake of BH3I-1 was measured by colorimetric detection of drug-associated bromine in cell lysates (Sollo et al., 1971).

**Statistical analysis**

Each experiment was performed at least three times and statistical analysis was performed using a two-tailed Student's t-test. Multiple comparisons were performed using ANOVA test and post-hoc Bonferroni correction was applied. Statistical probabilities (p) were expressed as *, where p<0.05, ** – p<0.01 and *** – p<0.001.
